# GPs’ experiences with brief intervention for medication-overuse headache: a qualitative study in general practice

**DOI:** 10.3399/bjgp14X681313

**Published:** 2014-09-01

**Authors:** Jan C Frich, Espen Saxhaug Kristoffersen, Christofer Lundqvist

**Affiliations:** Institute of Health and Society, University of Oslo, Oslo, Norway.; Department of General Practice, Institute of Health and Society, University of Oslo, Oslo; HØKH, Research Centre, Akershus University Hospital, Lørenskog, Norway.; HØKH, Research Centre, Akershus University Hospital, Lørenskog; Institute of Clinical Medicine, Campus Akershus University Hospital, University of Oslo, Nordbyhagen; Department of Neurology, Akershus University Hospital, Nordbyhagen, Norway.

**Keywords:** headache disorder, secondary, analgesic/adverse effects, primary care, qualitative study

## Abstract

**Background:**

Medication-overuse headache (MOH) is common in the general population, and most patients are managed in primary health care. Brief Intervention (BI) has been used as a motivational technique for patients with drug and alcohol overuse, and may a have role in the treatment of MOH.

**Aim:**

To explore GPs’ experiences using BI in the management of patients with MOH.

**Design and setting:**

Qualitative study in Norwegian general practice.

**Method:**

Data were collected through four focus group interviews with 22 GPs who participated in an intervention study on BI for MOH. Systematic text condensation was used to analyse transcripts from the focus group interviews.

**Results:**

The GPs experienced challenges when trying to convince patients that the medication they used to treat and prevent headache could cause headache, but labelling MOH as a diagnosis opened up a space for change. GPs were able to use BI within the scope of a regular consultation, and they thought that the structured approach had a potential to change patients’ views about their condition and medication use. Being diagnosed with medication overuse could bring about feelings of guilt in patients, and GPs emphasised that a good alliance with the patient was necessary for successful change using BI to manage MOH.

**Conclusion:**

GPs experience BI as a feasible strategy to treat MOH, and the technique relies on a good alliance between the doctor and patient. When using BI, GPs must be prepared to counter patients’ misconceptions about medication used for headache.

## INTRODUCTION

Medication-overuse headache (MOH) is one of the most common chronic headaches with a prevalence of 1–2% in the general population.^1^–^3^ The International Classification of Headache Disorders (ICHD) describes MOH as a:

*‘... headache occurring on 15 or more days per month developing as a consequence of regular overuse of acute or symptomatic headache medication (on 10 or more, or 15 or more days per month, depending on the medication) for more than 3 months. It usually, but not invariably, resolves after the overuse is stopped*’.*4*

Medication causing the symptoms could be regular analgesics, ergotamines, triptans, opioids, or a combination of medications.

MOH is a heterogeneous disorder, which has been suggested to include subgroups with simple medication overuse as well as complex cases with more ‘dependency-like’ behaviour.^5^–^7^ Research suggests that individuals with a ‘problem-solving mode’ with low acceptance of pain are more prone to developing MOH.^8^ In the general population, MOH can be identified through screening for headache frequency, followed by a short screening instrument for behavioural dependence, the Severity of Dependence Scale (SDS).^9^ Most MOH patients have consulted their GP for headache.^10^ It is well established that withdrawal of the overused medication in most cases leads to improvement of the headache, but treatment is challenging and varies from simple advice to lengthy in-hospital treatments.^1^,^11^ Patient education is an important part of the management of patients with MOH, and early recognition of patients with MOH and patients at risk for MOH is recommended as a clinical strategy.^11^,^12^

Screening and brief intervention (BI) is a well-known approach to identify and treat overuse of alcohol and other addictive drugs.^13^–^15^ BI involves the use of an identification tool followed by feedback to the identified individual as being ‘at risk’. The final step is to give information suggesting reduction of the use of the particular substance. A review identified several barriers to implementation of screening and BI for alcohol problems in general practice, such as lack of time, resources, and training. Context and timing was also an issue.^16^ Although there is a considerable amount of research exploring screening and BI for alcohol overuse,^16^ there are no previous studies on GPs’ experiences using BI for MOH. Studies of factors that influenced GPs’ views on screening and BI for alcohol problems in Denmark and Norway found that integration of screening and BI into existing routines could be difficult, and that screening and BI could represent a challenge to the relationship between the GP and patient.^17^,^18^

In the Brief Intervention for Medication-Overuse Headache (BIMOH) study, BI was adopted for managing MOH in general practice, and a pragmatic cluster-randomised controlled trial (RCT) was conducted to test BI against business as usual (Box 1).^19^

Box 1. **The Brief Intervention for Medication-Overuse Headache (BIMOH) study**

The study was a double-blind, pragmatic cluster randomised parallel controlled trial in primary care in Norway. Outcomes were based on interviews with patients who were assessed 3 and 6 months after the intervention. The participating GPs received a 1-day course held by headache specialists. Patients recruited by screening of participating GPs' patient lists were cluster randomised and received treatment by their GP. A total of 25 486 patients were screened, responder rate was 42%, A random sample of 104 who screened positive for possible MOH were invited, 73 of these participated. GPs in half of the continuous medical education (CME) groups received the BI course initially and used BI to manage their own patients, while the others ran their clinical business as usual (BAU). After 6 months, the GPs in the BAU group also received the BI course as a part of the design and thereafter applied the BI to their listed patients (initially BAU group). The BI method consisted of GPs first evaluating their MOH patients using the Severity of Dependence Scale. Based on this, the patients received feedback about the personal risk of MOH, and recommendations for reducing intake of headache medication. In all, 18 CME groups were invited, of whom three CME groups did not respond to the invitation and five groups declined participation. Thus, 10 CME groups with 50 GPs were included. Based on national figures the included GPs were representative in terms of practice localisation (urban versus suburban) and age distribution, but there were more female GPs among participants in the sample.

**How this fits in**

Medication-overuse headache (MOH) is a cause of chronic headache in the general population, but structured, evidence-based instruments for handling MOH have not, so far, been generally available. Brief intervention (BI) may be such an instrument. This study suggests that GPs experience BI as a feasible strategy to treat MOH in general practice. The results underscore that using BI requires a good alliance between the doctor and patient, and GPs must be prepared to invest effort into countering patients’ misconceptions about medication.

Results from the study suggest that BI performed by GPs, after a 1-day training course, reduces headache days and medication use in patients with MOH.^20^

### Aim

As doctors with a background in general practice, neurology, and health services research, the authors had an interest in learning more about GPs’ experiences of using the BI technique for MOH, as part of a process evaluation^20^ of the BIMOH study.^21^ This study was therefore performed to explore GPs’ experiences using BI in the management of patients with MOH.

## Method

### Participants

A qualitative study, using focus group interviews to collect data, was considered to be a suitable design. Focus groups were chosen to allow for exploration of group experiences and dynamics. Four focus group interviews were conducted with 22 GPs (nine males and 13 females), mean age 52.5 years (range 42–67 years). A purposeful sampling strategy was used to recruit GPs in continuous medical education (CME) groups from a total of 10 CME groups and 50 GPs who participated in the BIMOH study. The aim was a sample that represented variation regarding geography, age, and sex. All GPs gave written consent to participate in the focus group interview study. As all members of CME groups were invited to participate in the study, and patients were subsequently identified by screening of participating GPs' patient lists, the number of patients recruited per GP varied, and some GPs had no patients. All participating GPs received BI training. Sixteen of the 22 GPs had used the BI protocol on their patients (one on four patients, three on three patients, seven on two patients, and five had performed BI on one patient each). GPs were approached through the group coordinator by phone or email, and none of the GPs who were invited declined to participate.

### Brief intervention

GPs were taught to perform a structured BI for patients with possible MOH. The patients were identified based on a population screening of their patient lists. The GP first scored each patient individually using a 5-item interview based on the SDS. Based on the individual SDS result GPs used a structured scheme and a flip-over presentation to inform patients about:

- their SDS score and the associated risk for MOH based on previous results from the general population;^9^
- the need, therefore, to cut down on medication use;
- the expected gains and difficulties to be overcome; and
- how the withdrawal could be achieved with the support of the GP.

**Figure 1.**
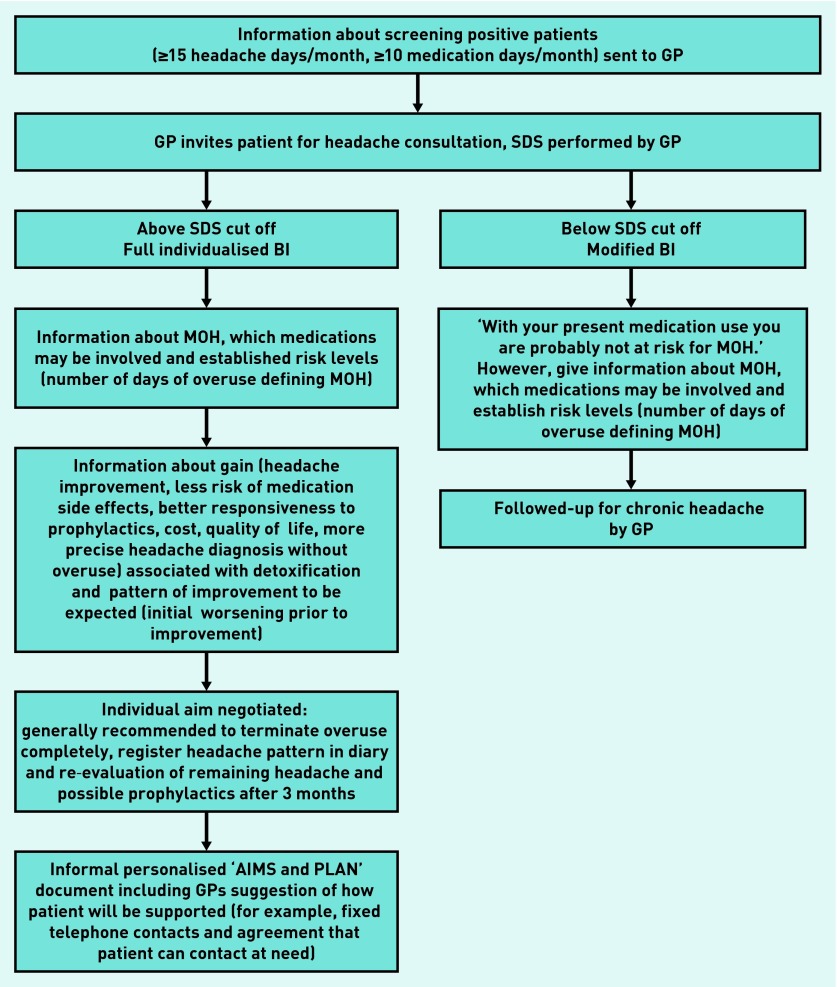
***Clinical workflow for the Brief Intervention for Medication-Overuse Headache (BIMOH) study. SDS = Severity of Dependence Scale.***

Feedback from a sample of GPs during a separate small pilot study of the logistics of BI suggested that the procedure took an average of 9 minutes to complete in one single clinical consultation. The information given included relating the individual SDS score to established cut-offs for MOH,^9^ that a worsening of headache over 1–2 weeks could be expected before improvement, and that gains were a good chance of improved headache and of improved responsiveness to prophylactics and the possibility of being prescribed these, should the headache not improve. Figure 1 illustrates the clinical workflow in the BIMOH study.

### Focus group interviews

Two of the authors jointly moderated three focus group interviews, and one of these was sole moderator for the fourth group. The focus groups interviews were conducted in January 2013, lasted 35–45 minutes, and were digitally recorded. The moderators used a thematic interview guide that covered experiences of using BI. Among specific questions that were discussed were: was using brief intervention as part of an ordinary consultation feasible? What resistance did you encounter in patients? What factors do you experience as effective? Have you used similar strategies in other patients? Six of the GPs had not used the protocol on their patients, but were active in the discussion, commenting on their colleagues’ experiences using BI.

### Analysis

The audio files were transcribed verbatim, and the written material was subject to analysis. The analysis followed the principles of systematic text condensation^22^ as follows: reading all the material to obtain an overall impression and bracketing previous preconceptions; identifying units of meaning, representing different aspects of the theme and coding for these; condensing and summarising the contents of each of the coded groups; and generalising descriptions and concepts about the specific theme. One author did the preliminary analysis, and the other authors participated in the final analysis. Illustrative quotes have been translated from Norwegian.

## RESULTS

Three main themes emerged from the analysis: medication use and resistance to change, feasibility using BI, and views on how BI works. These themes are elaborated further below.

### Medication use and resistance to change

GPs experienced that patients categorised medication as ‘problem-solvers’, and that patients’ previous experiences with pain relief influenced their assumptions that medication used to treat headache could not cause pain. A common misconception among patients was that medications such as paracetamol and NSAIDs were harmless, as they could be bought over the counter without being prescribed by a doctor, as illustrated in this quote:

*‘Patients do not see this as dangerous medication, because they can buy it anywhere. They can buy it by themselves when they need to. So, if it had been dangerous medication* […] *one would need a prescription.’*

(Focus group 2)

GPs stated that patients often mentioned anxiety for headache as a reason for using medication, and some patients had started using medication routinely as a preventive measure to avoid getting headache:

*‘You get the impression that people use too much because they are afraid of getting* [headache]*.’*

(Focus group 1)

Some patients reported to GPs that they needed to take medication to go to work. Faced with these views and symptoms similar to dependency, GPs experienced challenges conveying the message that medication could cause headache, as underlined by a GP:

*‘*[Patients] *say: ‘Yes, but I had headache before I started using these pills’. You have to spend some time on this, you cannot just state that this is how it is.’*

(Focus group 1)

GPs reported that specialist health care, in some cases, could legitimise the overuse and create or worsen dependency-like behaviour, making it even more difficult for a GP to argue that the medication also could cause pain, as noted by one participant:

*‘When they have received blessings from the pain clinic, well, then* [the patients] *have good arguments to put forward.’*

(Focus group 3)

As the mechanism of MOH and dependency was not evident to patients, GPs experienced that one needed to use time to inform the patient and to clarify misunderstandings.

### Feasibility of brief intervention

The GPs experienced using brief intervention for MOH as a feasible approach, and in most cases it was possible for them to do the intervention within a regular consultation. The GPs emphasised that they had to individualise the information to different patients:

*‘This is behavioural stuff, so it requires that one is able to reason, in my opinion, to be able to understand how things are connected* […] *you may have to adapt the information, to modify it, but I think it can be useful in all* [patients]*.’*

(Focus group 2)

Reasons for why a GP chose not to use BI could be language barriers or multimorbidity, as patients used analgesics for other medical conditions, such as rheumatism. The GPs also argued that in some patients the motivation to make changes could be low or lacking, because people had difficulties in their lives that caused more urgent concerns. Some GPs pointed out that the sample of patients in the study was self-recruited, and that their experiences may not be valid for all patients:

‘I am convinced or I know that I have patients who would be suitable for this study, and who did not participate. The motivation is perhaps missing.’

(Focus group 1)

The population screening procedure in the study identified patients that the GPs would not think had MOH, and they were unsure how they could identify these patients in regular general practice.

### Views on how brief intervention work

Some GPs mentioned that explicitly labelling medication overuse as a diagnosis could relieve patients from guilt. Patients would tend to blame themselves, and the diagnosis created a space for action. GPs agreed that one had to give information without moralising. Some GPs thought that the systematic, pedagogical approach they used in BI was credible, and that it had the potential to change how patients perceived their condition and medication use, as emphasised by a GP:

*‘The good thing is that you learn, you do a kind of teaching session, so that they can understand, and you show them why they have this pain, why it happens, then something happens with* [their] *perception of the pain.’*

(Focus group 4)

Using written material and the flip-over was reported by participants as an efficient and appropriate way of informing the patient:

*‘The flip-over … it was easy, with few points, and they were clear, and this was new to those who came. It was good, and in my experience they have absorbed the points, all of them* […] *It was efficient.’*

(Focus group 2).

The fact that the message was clear and easy to understand was emphasised by GPs:

‘To get some clear messages, that is what I think is good about this in a way, make it simple, make it clear. Well, it has to be simple; it has to be an easy message’

(Focus group 1).

The GPs said that using the flip-overs written by other authoritative health professionals allowed them to tell the patient that the knowledge was new for them too, and that this could strengthen the alliance with the patient. They underlined that respect and a good alliance with the patient was important for successful change. Acknowledging the patients’ symptoms was also emphasised by the GP as fundamental when using BI:

‘I think it is extremely important to acknowledge that it is painful to have pain. To let, in a way, that respect and, yes, that caring, follow the advice to reduce the use of medication.’

(Focus group 1).

Letting the patient talk about their concerns was underscored by GPs as important:

*‘To be taken seriously* […] *to get time, to be allowed to talk about what you struggle with — because what they have is a problem. I think that works.’*

(Focus group 4)

Some GPs argued that taking part in a research project, and being examined by a specialist, probably influenced patients’ motivation for change.

## DISCUSSION

### Summary

It was found that GPs experienced challenges when trying to convince patients that the medication they used to treat and prevent headache could cause headache, but labelling MOH as a diagnosis opened up a space for change. GPs were able to use BI within the scope of a regular consultation, and they thought that the structured approach had a potential to change patients’ views about their condition and medication use. Being diagnosed with medication overuse could bring about feelings of guilt in patients, and GPs emphasised that a good alliance with the patient was necessary for successful change using BI to manage MOH.

### Strengths and limitations

Participating GPs attended a 1-day educational course to learn about MOH and BI, which was part of the BIMOH study. As a group, they are more experienced than the average GP in Norway, and the sample may not be fully representative. The patients had been identified as being at risk of MOH in a population survey, and had consented to participate in a study, but medication overuse was not a theme in the invitation. According to previous results,^2^ it can be assumed that responders of the screening questionnaire were reasonably representative. The intervention group and the control group were also comparable. Those who responded to BI and improved may, however, be a selection of responsive individuals. Their motivation and resources to do something with their headache may be higher than for the average patient. Still, this does not undermine that BI seems to be a feasible approach for managing patients with MOH in general practice.

### Comparison with existing literature

It is well documented that motivating patients to change health behaviours represents a challenge for health professionals,^23^ and previous research suggests that patients with MOH are inclined to hold on to what they consider to be ‘indispensable medication’.^24^ In MOH, patients’ beliefs about medication represent a potential barrier to change, and this study documents specific misperceptions that GPs may encounter when managing patients with MOH. Previous research has documented barriers to using BI to identify and manage patients with alcohol overuse in general practice.^16^–^18^ The present study suggests that GPs find BI to be a feasible approach to managing patients with MOH, but may not recommend using the technique if there is a language barrier or if the patients use analgesics for a comorbid condition or have medical conditions calling for immediate attention. Trust, respect, and recognition are important in all doctor–patient relationships,^25^ and the present results underscore that BI could be used if the doctor has established a good alliance with the patient.

### Implications for research and practice

This study suggests that medical knowledge about the cause of MOH may contradict patients’ mental models of their headache and the drugs they use. Medication can have different symbolic meanings for patients. Helman demonstrated that users of psychotropic medication classified drugs as ‘tonic’, ‘fuel’, and ‘food’, and that these categories correlated with patterns of use.^26^ Some patients with MOH appear to use medication for headache not only as a means to relieve symptoms, but also to prevent an expected headache. The drugs have become ‘food’ that the patients think needs to be taken on a regular basis.

In clinical practice, GPs may not know that a patient is at risk of MOH. The diagnosis may be recognised when the patient presents a medical history and symptoms indicating MOH. Physicians may refrain from addressing dependency-like behaviours out of fear of provoking patients or for not having enough time to discuss such ‘complicated’ and sensitive issues. Simple, evidence-based instruments like BI may be a help when explaining how overuse of analgesics can cause dependency-like behaviour and headache.

This study suggests that a structured approach such as BI is feasible for managing patients with MOH in general practice. BI does not seem to provoke the patients or lead to physician–patient conflicts when it is used for MOH. Structural barriers, as reported in use of BI for alcohol problems, did not come up as a big issue in this study.^16^–^18^ Outside a study situation, a GP’s alliance with a patient over time may be an important additional factor for success of BI. However, this requires further studies and a prerequisite is that the GP is aware of the patient’s risk of MOH in advance. The BI training and increased knowledge of MOH in general may thus be an approach that holds promise for helping motivated patients in general practice. Less motivated patients and patients who do not respond to the BI may still require referral to specialist headache or neurological clinics, perhaps even to in-patient detoxification.

GPs experience BI as a feasible strategy to treat MOH in general practice, and the technique relies on a good alliance between the doctor and patient. When using BI, GPs must be prepared to invest effort to counter patients’ misconceptions about medication used for headache.
